# Protective effects of *Fragaria ananassa* methanolic extract in a rat model of cadmium chloride-induced neurotoxicity

**DOI:** 10.1042/BSR20180861

**Published:** 2018-11-16

**Authors:** Manal F. Elkhadragy, Rami B. Kassab, Dina Metwally, Rafa S. Almeer, Rewaida Abdel-Gaber, Ebtesam M. Al-Olayan, Ehab A. Essawy, Hatem K. Amin, Ahmed E. Abdel Moneim

**Affiliations:** 1 Department of Zoology, College of Science, King Saud University, Riyadh, Saudi Arabia; 2 Department of Zoology and Entomology, Faculty of Science, Helwan University, Cairo, Egypt; 3 Department of Parasitology, Faculty of Veterinary Medicine, Zagazig University, Zagazig, Egypt; 4 Zoology Department, Faculty of Science, Cairo University, Cairo, Egypt; 5 Department of Chemistry, Faculty of Science, Helwan University, Cairo, Egypt; 6 Department of Biochemistry and Molecular Biology, Faculty of Pharmacy, Helwan University, Cairo, Egypt

**Keywords:** apoptosis, brain, cadmium, Fragaria ananassa, oxidative stress

## Abstract

Cadmium (Cd) is a common environmental toxicant that has harmful effects on plants, animals, and humans. The present study evaluated the protective effects of *Fragaria ananassa* methanolic extract (SME) on cadmium chloride (CdCl_2_)-induced neuronal toxicity in rats. Male albino rats were intraperitoneally (i.p) injected with CdCl_2_ (6.5 mg/kg) for 5 days with or without the SME (250 mg/kg). We measured the levels of Cd, lipid peroxidation (LPO), nitric oxide, glutathione (GSH), and oxidative enzymes such as superoxide dismutase (SOD), catalase (CAT), glutathione peroxidase, and glutathione reductase (GR) in the whole brain homogenate. Compared with the control group, the Cd-intoxicated group showed a marked increase in the brain levels of Cd, LPO, and nitric oxide and a decrease in the levels of GSH and all tested antioxidant enzymes. Compared with Cd-intoxicated rats, the rats pretreated with SME showed restoration of oxidative balance in the brain tissue. While the expression of brain *SOD2*, CAT, glutathione peroxidase 1, and GR was down-regulated in the Cd-treated group, the expression of these enzymes was up-regulated in rats pretreated with SME. In addition, administration of SME before CdCl_2_ increased the Bcl-2 expression, but significantly decreased the expression of Bax. Immunohistochemical analysis showed that compared with Cd-intoxicated rats, rats pretreated with SME showed a decrease in the protein expression of tumor necrosis factor α (TNF-α). Our findings indicate that SME protects the brain tissue from Cd-induced neuronal toxicity by improving the antioxidant system and increasing antiapoptotic and anti-inflammatory activities.

## Introduction

Cadmium (Cd) is one of the most toxic heavy metals and a common industrial pollutant present in the environment [1,2]. The main sources of exposure to Cd are smoking tobacco, air pollution, and drinking contaminated water [3]. Additional sources include exposure through fertilizers, sewage, sludge, wastewater, pesticides, metal plating, pigments, plastics, glass, and batteries [4]. Cd is poorly excreted; Cd has a long biological half-life (20 years) and cannot be degraded, and thus accumulates in different organs of the body [5]. The bioaccumulation of Cd in the living system may cause severe damage to the reproductive system, gastrointestinal tract, mucous tissues, and nervous system [6]. Cd inhaled though the nasal mucosa or olfactory pathways enters the central nervous system (CNS) thus causing neurotoxicity [7]. The neurotoxicity of Cd has been observed in cultured rat cortical neurones [8] and rat primary midbrain neurone-glia cultures [9]; Cd disturbs the normal neurochemistry of the animal brain [10,11]. The mechanisms of Cd-induced neurotoxicity have not been clarified thus far. Oxidative stress has been proposed as a possible mechanism for Cd toxicity in a number of tissues such as the kidney [12], liver [2,13], and brain [14]. Cd is indirectly involved in the generation of reactive oxygen species (ROS) via the inactivation of thiol groups in critical molecules, inhibiting antioxidant defenses and DNA repair mechanisms, and disturbing the antioxidant system [15].

The use of herbal plants as health promoters is gaining increasing popularity in the consumer market and in the scientific circle [16,17]. Fruits and vegetables have been used as medicines for a long time because of they are economical and have few side effects on animals and humans [18]. They are important sources of vitamins, minerals, antioxidants, and phytochemicals [19].

Strawberry (*Fragaria ananassa*) is one of the most consumed fruits in the world. The major active ingredients of this fruit include the vitamin β-carotene and phenolic compounds such as phenolic acids, flavonoids, and anthocyanins [20]. Our group previously demonstrated that the methanolic extract of strawberry contains many active ingredients such as gallic acid, *p*-coumaric acid, cyanidin, pelargonidin, ellagic acid, and quercetin. Furthermore, the extract has high concentration of total phenolics content ranged from 98.3 to 112.7 mg/g extract, total flavonoids content ranged from 44.2 to 57.1 mg/g extract, and the total anthocyanin content ranged from 12.6 to 25.3 mg/g extract [21]. These bioactive compounds provide health benefits through several mechanisms, including the scavenging of ROS, protecting and regenerating other dietary antioxidants, and chelation of pro-oxidant metals [22]. These active ingredients have various pharmacological activities such as prevention of inflammation, hepatotoxicity, cancer, obesity, and oxidative stress [21,23]. The antioxidant properties of strawberry enable its use in models in which oxidative stress might play a role; therefore, in the present study, we investigated the effect of *F. ananassa* methanolic extract (SME) on Cd induced-neurotoxicity in rats.

## Materials and methods

### Chemicals

Cadmium chloride (CdCl_2_) anhydrous was purchased from Sigma Chem. Co. (St. Louis, MO, U.S.A.). Tris/-HCl was obtained from Fluka Chemie (Buchs, Switzerland). Thiobarbituric acid was obtained from Merck (Darmstadt, Germany), nitroblue tetrazolium and phenazine methosulphate were purchased from Alfa Aesar (Tewksbury, MA, U.S.A.).

TRIzol isolation kit was obtained from Invitrogen (Carlsbad, CA, U.S.A.). RevertAid H minus Reverse Transcriptase was purchased from Thermo Fisher Scientific Inc. The PCR primers were synthesized by Jena Bioscience GmbH (Jena, Germany). All other routine chemicals and solvents were of pure analytical grade.

### Fruit material

*F. ananassa* fresh fruit was purchased from a local market in Cairo, Egypt in April–May 2016. The plant specimen was identified by the Botany Department, Faculty of Science, Helwan University. Fruits were homogenized as 1:10 w/v of 70% methanol aqueous solution. Homogenate was filtered out and left to dry using a vacuum evaporator (IKA, Germany). The residues were dissolved in distilled water and stored in an air-tight bottle at −20°C and was labeled as SME.

### GC-MS analysis

The GC-MS analysis of SME was performed with a Thermo Scientific, Trace GC Ultra & ISQ Single Quadruple MS. Helium (99.999%) was used as the carrier gas with a flow rate at 1.7 ml min^−1^.1 μl injected (1:10) using the splitless injection technique; Agilent 19091S-433UI column (HP-5ms Ultra Inert GC Column, 30 m, 0.25 mm, 0.25 µm, 7-inch cage). Temperatures: injector: 250°C, detector: 280°C, column: 100°C with a temperature gradient of 20°C.min^−1^, 260°C held for 10 min. The total GC running time is at 20 min. The MS ionization voltage was 70 eV. The MS scan parameters included a mass range of m/z 40–1000 atomic mass units (amu). Identification of compounds was matched using the database of Wiley9, replib, and mainlib Libraries. The name, retention time, and area under peak of the components of the test materials were ascertained.

### Animal treatment

To evaluate the potential neuroprotective effects of SME on CdCl_2_-intoxicated rats, we used 32 adult male Wistar rats, 35–42 days old, with an initial body weight of 160–180 g. The animals were obtained from the Holding Company for Biological Products and Vaccines (VACSERA, Cairo, Egypt). The rats were housed in polypropylene cages and were maintained at room temperature (22 IS ± 3°C) on 12-h light/12-h dark cycle throughout the experiment. The rats were supplied water *ad libitum* and fed with a commercial pelleted rat chew diet of standard quality. They were allowed to adapt to the laboratory conditions for 7 days before starting the experiment. Animal care was in accordance with the National Institutes of Health (NIH) Guidelines for the Care and Use of Laboratory Animals 8th edition (NIH Publication No. 85-23, revised 1985) and the study protocol and animal handling procedures were approved by the Ethical Committee of the Faculty of Pharmacy, Helwan University under approval number 0015A-16.

The animals were randomly divided into four equal groups (*n*=8) as follows:

1. Control group (Con): the rats were injected daily intraperitoneally (i.p) with normal saline for 5 days.
2. CdCl_2_-intoxicated group: the rats were injected i.p with 6.5 mg/kg CdCl_2_ for 5 days according to the procedure described by Dkhil et al. [13].
3. SME group: animals were orally administered SME (250 mg/kg) daily for 5 days.
4. Combined treatment group (SME + CdCl_2_): animals were pretreated with SME orally, then after 1 h, they were injected i.p with CdCl_2_ for 5 days.

The animals were killed by sudden decapitation 24 h after the last treatment, their brains were rapidly excised from the skulls, blotted, and chilled. The brain tissue was rapidly wiped dry using filter paper and homogenized in ice-cold medium of 50 mM Tris/HCl (pH 7.4) to give a 10% (w/v). The total protein content of the homogenized brain was then determined using the standard method of Lowry et al. [24].

### Cadmium concentration

Cadmium concentration in the brain samples was quantitatively estimated after the end of the experiment using an atomic absorption spectrophotometer at 228.8 nm according to the protocol described by Kubaszewski et al. [25]. The tissue sample was weighed and ashed in a muffle furnace at 500°C, and then digested using nitric acid at 150°C for 2 h. The digested sample was diluted with deionized water to 50 ml. The level of cadmium was expressed as ng/g wet tissue.

### Biochemical assay

#### Estimation of oxidants

The level of malondialdehyde (MDA) was estimated as a lipid peroxidation (LPO) marker in the brain tissue using the method described by Ohkawa et al. [26]. Briefly, a mixture consisting of distilled water, 0.67% thiobarbituric acid, and 0.22% sulphuric acid was added to 500 µl of brain supernatant. The formed mixture was boiled at 95°C for 30 min and then cooled at room temperature and centrifuged for 15 min at 1000 ***g***. Finally, the supernatant was estimated spectrophotometrically 540 nm. The obtained data are expressed as nanomoles MDA per milligram of protein. Nitric oxide (NO) level was measured using the Griess reagent according to Green et al. [27]. Briefly, 100 μl of brain supernatant was mixed for 10 min with Griess reagent at room temperature. The formed reddish purple azo dye was measured spectrophotometrically at 540 nm.

#### Estimation of antioxidants

Glutathione (GSH) was estimated by the reduction of Elman’s reagent (5,5′-dithiobis (2-nitrobenzoic acid) (DTNB)) with GSH to produce a yellow compound. The reduced chromogen is directly proportional to the GSH concentration, and its absorbance can be measured at 405 nm [28]. Catalase (CAT) activity was determined as described by Aebi [29]. Briefly, 0.1 ml of the brain homogenate was mixed with mixture containing phosphate buffer (50 mM, pH 7.0) and H_2_O_2_ (30 mM). The decrease in absorbance was observed for 3 min and the enzyme activity was determined as μM H_2_O_2_ decomposed/s/mg protein. The activity of superoxide dismutase (SOD) was determined using the method of Nishikimi et al. [30]. Briefly, 0.05 ml of brain homogenate was added to mixture containing phenazine methosulphate (93 µM), reduced NAD (0.47 mM), nitroblue tetrazolium (0.3 mM), and phosphate bufer (0.1 M; pH 8.5). The increase in absorbance was estimated at 560 nm for 5 min. The obtained results were expressed as units per milligram of protein. Meanwhile, glutathione peroxidase activity was measured using the method of Paglia and Valentine [31]. GPx activity was estimated in terms of the decrease in NADH per min using a reaction coupled with glutathione reductase (GR). The decrease in absorbance at 340 nm was recorded, and GPx activity was expressed as U/mg protein. In addition, GR activity was measured by quantitating GSH-dependent oxidation of NADPH at 340 nm and expressed as U/mg protein.

### Quantitative reverse-transcriptase PCR analysis

Total brain tissue RNA was extract from frozen samples using the TRIzol reagent. Approximately 5 μg of total RNA was converted into cDNA. The Power SYBR® Green Master Mix kit was used for real-time PCR analysis. The reaction program was set as follows: 95°C for 30 s followed by 42 cycles at 94°C for 5 s and 58°C for 60 s. The relative differences in gene expression between groups were expressed using cycle time (*C*_t_) values, and the relative differences between groups were expressed as relative increases setting control as one fold. The glyceraldehyde-3-phosphate dehydrogenase (*GAPDH*) gene was used as a control, and the expression of *GAPDH* was unchanged by any treatment used. The sense and antisense primers for the selected gene sets used are as follows:

*GAPDH* (accession number: NM_017008.4; product length: 248): S: 5′-AGTGCCAGCCTCGTCTCATA-3′; AS: 5′-GATGGTGATGGGTTTCCCGT-3′ [32].

*SOD2* (accession number: NM_017051.2; product length: 191): S: 5′-AGCTGCACCACAGCAAGCAC-3′; AS: 5′-TCCACCACCCTTAGGGCTCA-3′ [32].

*CAT* (accession number: NM_012520.2; product length: 362): S: 5′-TCCGGGATCTTTTTAACGCCATTG-3′; AS: 5′-TCGAGCACGGTAGGGACAGTTCAC-3′ [32].

*GPx1* (accession number: NM_030826.4; product length: 245): S: 5′-CGGTTTCCCGTGCAATCAGT-3′; AS: 5′-ACACCGGGGACCAAATGATG-3′ [32].

*GR* (accession number: NM_053906.2; product length: 229): S: 5′-TGCACTTCCCGGTAGGAAAC-3′; AS: 5′-GATCGCAACTGGGGTGAGAA-3′ [32].

*Bcl-2* (accession number: NM_016993.1; product length: 228): S: 5′-CTGGTGGACAACATCGCTCTG-3′; AS: 5′-GGTCTGCTGACCTCACTTGTG-3′ [32].

*Bax* (accession number: NM_017059.2; product length: 217): S: 5′-GGCGAATTGGCGATGAACTG-3′; AS: 5′-ATGGTTCTGATCAGCTCGGG-3′ [32].

### Preparation of histological sections

The brains were removed and fixed in 10% neutral formalin for 24 h at room temperature and processed for paraffin sectioning at a thickness of 5 μm. The slides were stained with Hematoxylin and Eosin stain (H&E) according to the previously described method of Bancroft and Gamble [33] for general examination.

### Immunohistochemistry analysis

Formaldehyde/PBS-fixed, paraffin-embedded sections (4-μm thick) were mounted on glass slides. Sections were deparaffinized, blocked with methanol containing 0.1% hydrogen peroxide (H_2_O_2_) for 10 min to quench the endogenous peroxidase activity. After blocking, the sections were stained with polyclonal rabbit anti-tumor necrosis factor α (TNF-α) antibody at 4°C overnight. Then, the sections were washed with phosphate buffer and incubated with horseradish peroxidase–conjugated goat anti-rabbit antibody at 37°C for 30 min. The antibody-binding sites were visualized by incubation with diaminobenzidine (DAB)-H_2_O_2_ at room temperature for 10 min [34]. Images were taken at original magnification of 400× (Nikon Eclipse E200-LED, Tokyo, Japan).

### Statistical analysis

Statistical Package for the Social Sciences (SPSS) was used for data analysis. The results were expressed as the mean ± S.E.M. One-way ANOVA followed by Duncan’s test was applied for determining the significance. The acceptable level of significance was established at *P*<0.05.

## Results

### GC-MS results

**Figure 1:**
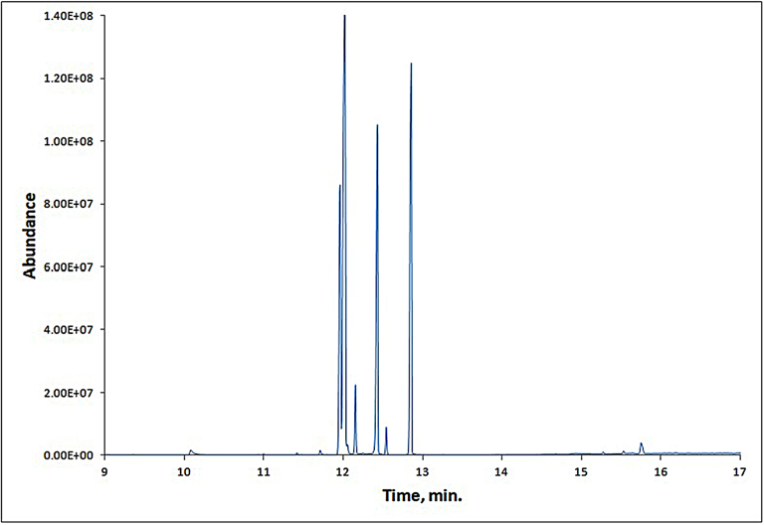
GC-MS chromatogram of SME

GC-MS chromatogram of the strawberry fruit extract (Figure 1) showed 41 peaks indicating the presence of 41 compounds. On comparison of the mass spectra of the constituents with Wiley9, replib, and mainlib libraries, the 41 compounds were characterized and identified (Supplementary Table S1). The identified compounds indicated the presence of many compounds that are considered as precursors for compounds responsible for strawberry fruits aroma and flavor besides other phytochemicals [35].

### Strawberry attenuates cadmium accumulation in brain tissue

**Figure 2:**
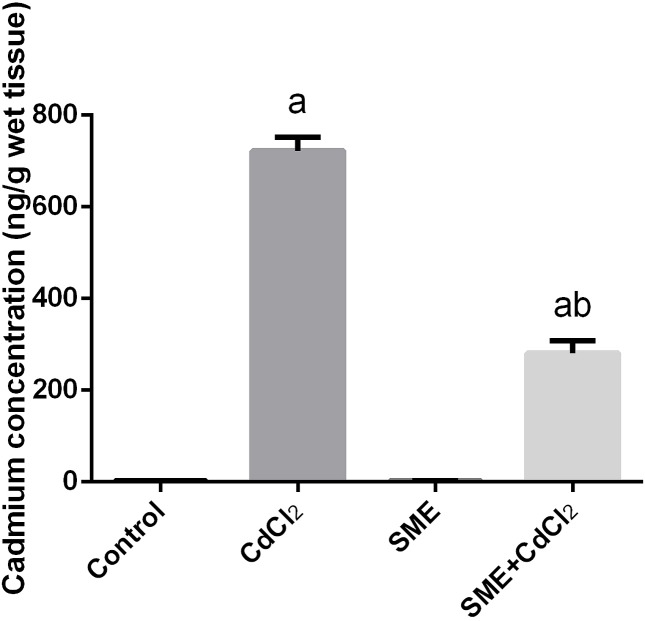
Neuroprotective effects of SME on cadmium accumulation (ng/g wet tissue) in the brain homogenate of rats exposed to CdCl_2_ for 5 days Data are expressed as the mean ± S.E.M. (*n*=8). ^a^*P*<0.05, significant change with respect to control; ^b^*P*<0.05, significant change with respect to CdCl_2_ using Duncan’s post-hoc test.

The Cd levels in the whole brain were significantly higher than normal levels (*P*<0.05) in rats injected with CdCl_2_ (700 ng/g wet tissue), this increase was significantly inhibited by treatment with SME (Figure 2).

### Strawberry attenuates cadmium alerted oxidant/antioxidant status

**Figure 3:**
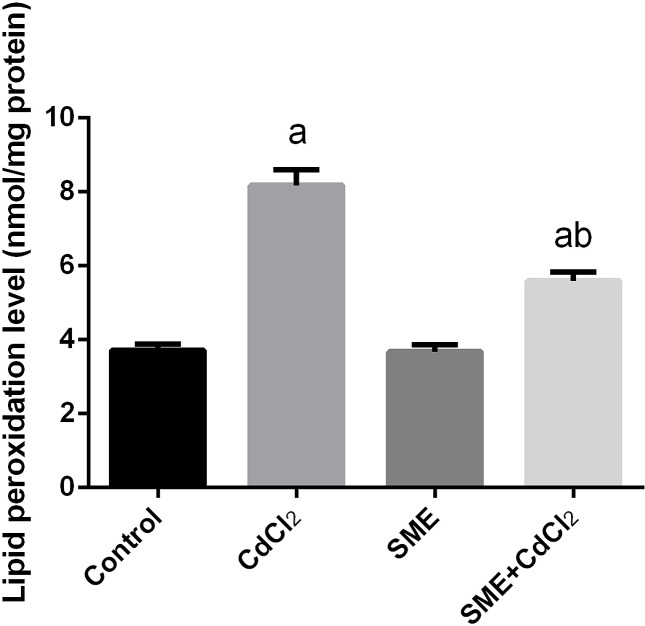
Ameliorative effects of the pretreatment with SME on the LPO level in rats intoxicated with CdCl_2_ for 5 days Data are expressed as the mean ± S.E.M. (*n*=8). ^a^*P*<0.05, significant change with respect to control; ^b^*P*<0.05, significant change with respect to CdCl_2_ using Duncan’s post-hoc test.

**Figure 4:**
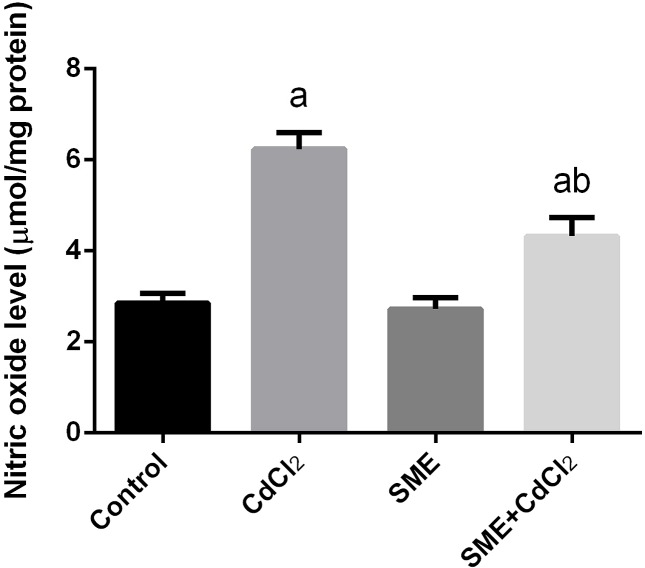
Ameliorative effects of the pretreatment with SME on the NO level in rats intoxicated with CdCl_2_ for 5 days Data are expressed as the mean ± S.E.M. (*n*=8). ^a^*P*<0.05, significant change with respect to control; ^b^*P*<0.05, significant change with respect to CdCl_2_ using Duncan’s post-hoc test.

**Figure 5:**
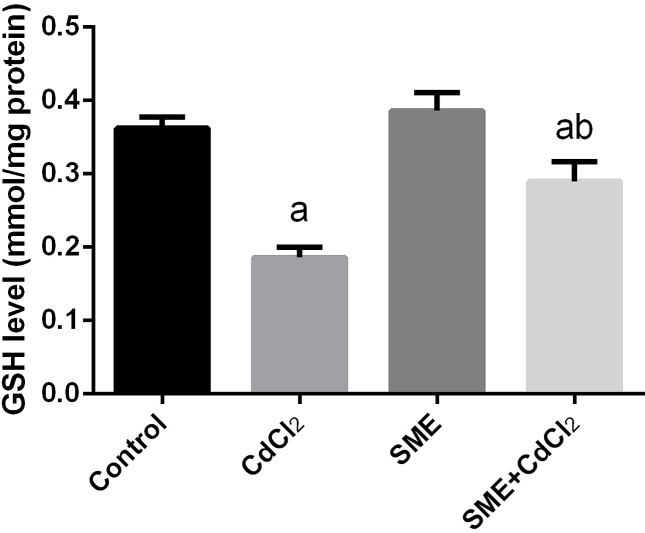
Ameliorative effects of the pretreatment with SME on GSH content in rats intoxicated with CdCl_2_ for 5 days Data are expressed as the mean ± S.E.M. (*n*=8). ^a^*P*<0.05, significant change with respect to control; ^b^*P*<0.05, significant change with respect to CdCl_2_ using Duncan’s post-hoc test.

Compared with the control group, the group exposition to CdCl_2_ injection showed an increase in the levels of LPO and nitric oxide (NO) and a significant decrease in the GSH levels (*P*<0.05) thus indicating an impairment in the oxidative balance in the brain tissue. The rats treated with a combination of CdCl_2_ and SME showed restoration of these levels to the control levels (*P*<0.05) (Figures 3–5).

**Figure 6:**
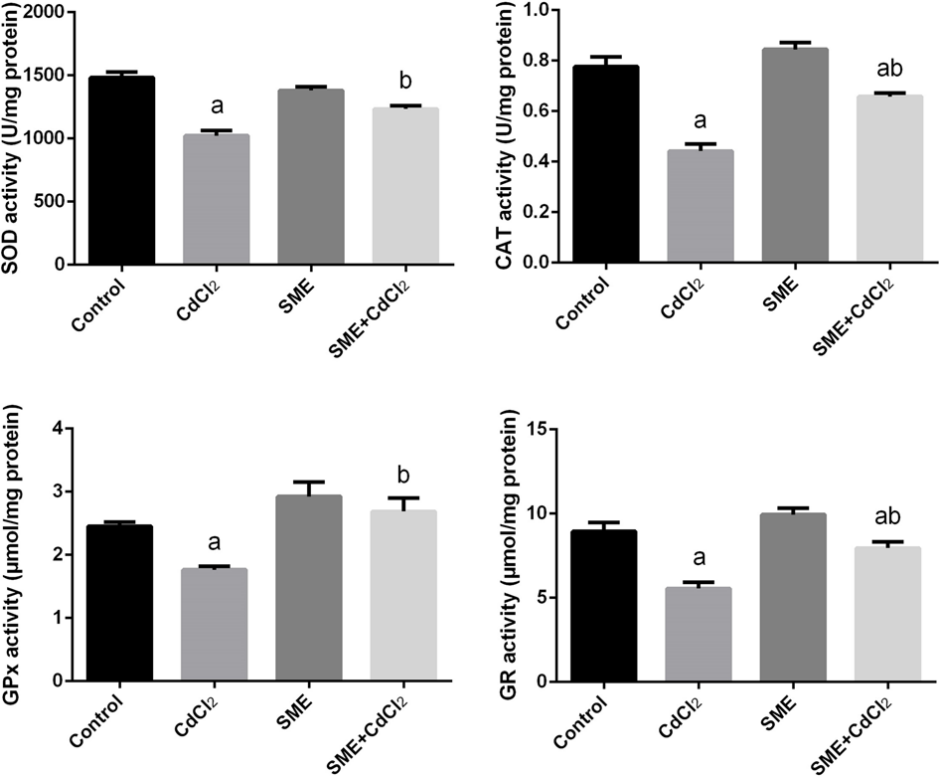
Ameliorative effects of the pre-administration of SME on the antioxidant enzyme activities in the brain tissue of rats exposed to CdCl_2_ for 5 days Data are expressed as the mean ± S.E.M. (*n*=8). ^a^*P*<0.05, significant change with respect to control; ^b^*P*<0.05, significant change with respect to CdCl_2_ using Duncan’s post-hoc test.

Our study showed changes in the activities of antioxidant enzymes namely SOD, CAT, GPx, and GR in the brain homogenate of the experimental rats (Figure 6). We observed a significant decrease (*P*<0.05) in the activities of SOD, CAT, GPx, and GR enzymes in CdCl_2_-intoxicated rats; moreover, compared with CdCl_2_-intoxicated rats, rats treated with SME showed an increase in the activities of these antioxidant enzymes.

**Figure 7:**
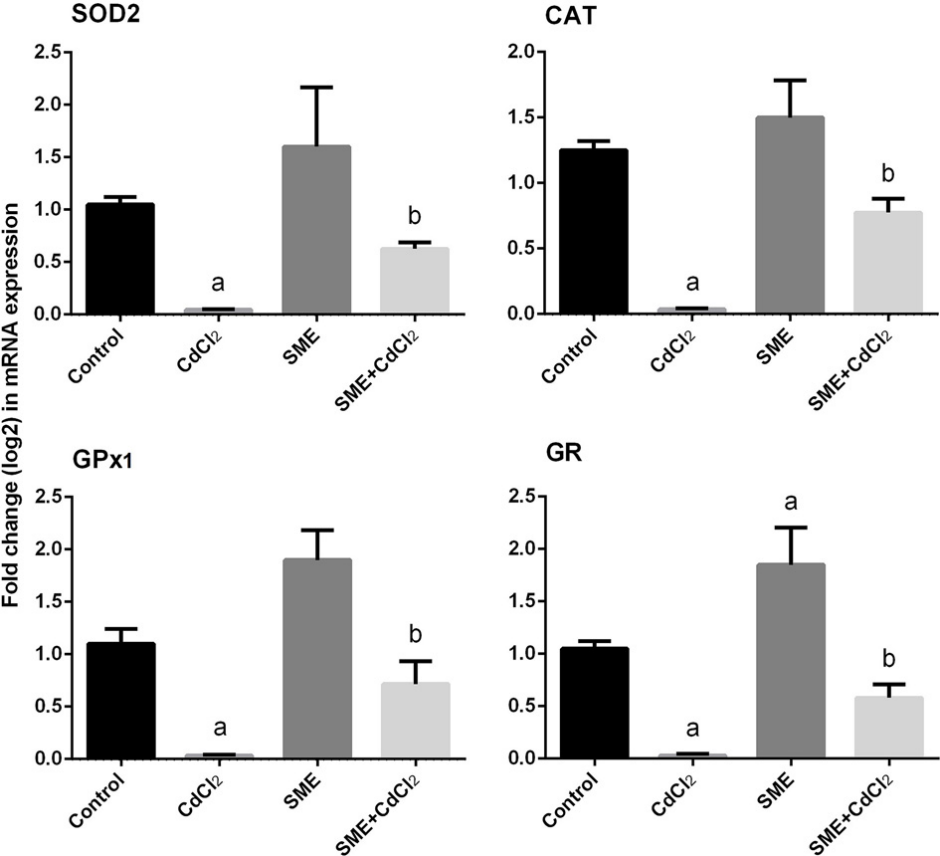
Ameliorative effects of the pre-administration of SME on the mRNA expression of antioxidant enzymes in the brain tissue of rats exposed to CdCl_2_ for 5 days Data of mRNA levels (mean ± S.E.M. of three assays) were normalized to *GAPDH* mRNA level and are shown as fold induction (in log_2_ scale) relative to the mRNA level in the control. ^a^*P*<0.05, significant change with respect to control; ^b^*P*<0.05, significant change with respect to CdCl_2_ using Duncan’s post-hoc test.

Exposition to CdCl_2_ significantly down-regulated (*P*<0.05) the mRNA expression of *SOD2, CAT, GPx1*, and *GR* with respect to their control values. Rats pretreated with SME showed a significant (*P*<0.05) increase in the expression of these antioxidant enzymes in the brain homogenate of CdCl_2_-intoxicated rats (Figure 7).

### Strawberry attenuates cadmium-induced apoptosis

**Figure 8:**
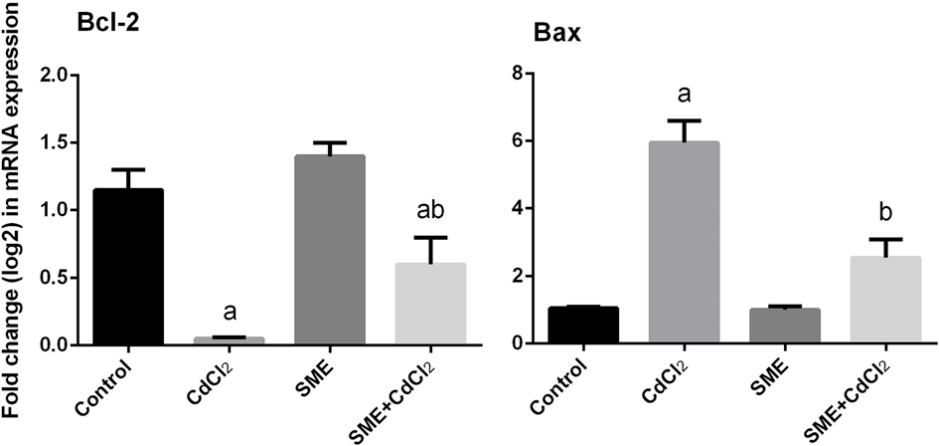
Mitigation effects of the pre-administration of SME on the mRNA levels of *Bcl-2* and *Bax* in the brain tissue of rats exposed to CdCl_2_ for 5 days Data of mRNA levels (mean ± S.E.M. of three assays) were normalized to *GAPDH* mRNA level and are shown as fold induction (in log_2_ scale) relative to the mRNA level in the control. ^a^*P*<0.05, significant change with respect to control; ^b^*P*<0.05, significant change with respect to CdCl_2_ using Duncan’s post-hoc test.

We determined the antiapoptotic activities of SME by examining the expression of *Bcl-2* and *Bax* in the brain tissue. qRT-PCR findings showed that the mRNA expression level of *Bcl-2* was down-regulated, whereas the *Bax* expression was up-regulated in CdCl_2_-treated rats. In addition, administration of SME before exposition to CdCl_2_ significantly alleviated the changes (*P*<0.05) in the levels of these proapoptotic markers (Figure 8).

### Strawberry attenuates cadmium-induced histopathological alternation

**Figure 9:**
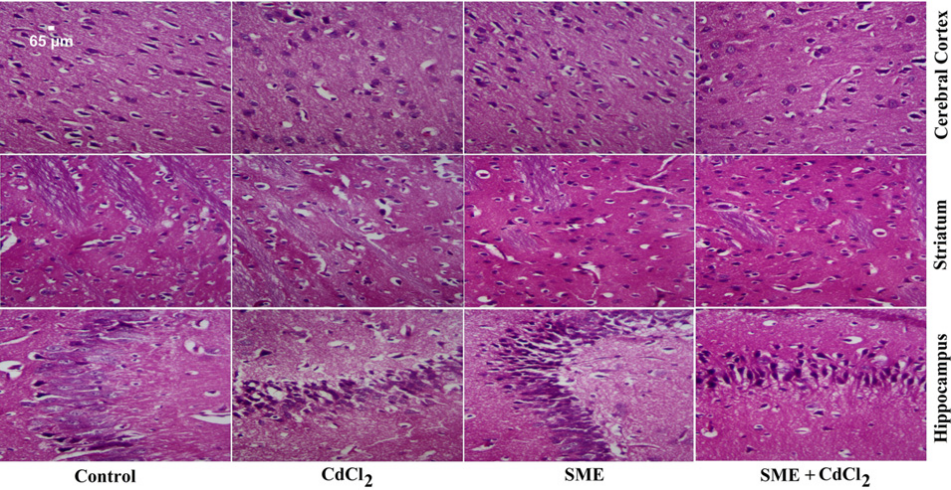
Effect of SME on the histological changes caused by CdCl_2_ injection in the brain of rats Sections of control rats show normal architecture in the different parts of brain. Sections from the CdCl_2_-intoxicated group show extensive neuronal damage, apoptotic neurones, and degeneration of Purkinje neurones. Sections of the SME-treated group show a normal structure. Sections of brains where SME was pre-administered to CdCl_2_ show improvement in the neuronal structure (H&E, ×400).

The brains of control rats exhibited their general characteristic shape. The nuclei of these neurones were normal, large, and centrally located (Figure 9). CdCl_2_-intoxicated rats showed marked damage to different regions of the brain, indicated by the degenerated and pyknotic nuclei in the hippocampus, degenerated, and pyknotic neurones in the striatum, and the presence of small shrunken cells in the cortex. Treatment with SME before administration of CdCl_2_ had protective effects on neurones, which suggested that the SME can alleviate neural injury induced by CdCl_2_ injection; however, despite pretreatment with SME, the morphology of some neuronal cells remained altered.

### Strawberry attenuates cadmium-induced inflammation

**Figure 10:**
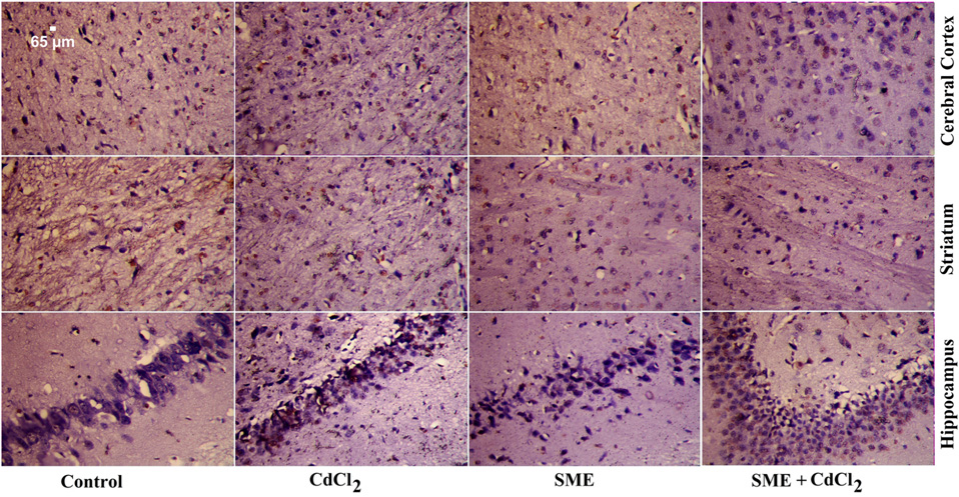
Immunohistochemical study of TNF-α in different regions of the brain Sections of the cerebral cortex, striatum, and hippocampus were obtained from control, CdCl_2_, SME, and SME + CdCl_2_-intoxicated rats. Few TNF-α-positive cells were observed in the control and SME groups. Increased TNF-α immunoreactivity was observed in CdCl_2_-intoxicated group. Moderate TNF-α immunoreactivity was observed in SME + CdCl_2_-treated group (×400).

We determined the anti-inflammatory activity of SME by measuring the protein expression of TNF-α. While the protein expression of TNF-α was significantly up-regulated in the CdCl_2_-intoxicated group (Figure 10), the group pretreated with SME showed a marked down-regulation of TNF-α in the brain homogenate.

## Discussion

To determine the biological activities of constituents present in the fruits and vegetables, the use of the whole extract is better than using individual compounds purified or isolated from the extract [36]. Recent studies have shown protective effects of strawberry extract, which contains high levels of phytochemicals, in different animal and cellular models of oxidative stress-induced diseases [37,38]. This study was performed to investigate the neuroprotective role of SME in Cd-induced neuronal toxicity in the brain tissue of male albino rats.

Cd is a toxic heavy metal that causes harmful effects on the cellular and metabolic systems in humans and animals. Our results showed that rats intoxicated with Cd had high levels of the metal in the brain tissue (approximately 700-fold of the control values) after 5 days of treatment. The accumulation of Cd in the brain tissue may be because of its ability to cross the blood–brain barrier [39]. Once inside, Cd accumulates in different brain tissue and causes cellular dysfunction [7,40]. Oral administration of SME was effective and markedly accelerated the clearance of Cd from the brain tissue after termination of exposure. The protective effects of SME may be attributed to the flavonoids content in the SME which sequester metal ions [41]. Previous reports showed that strawberry and its active constituents can cross BBB and may chelate Cd [42,43].

Cd neurotoxicity is due to the production of ROS, which leads to oxidative stress [44]. To confirm the mechanism underlying Cd neurotoxicity, we measured the levels of LPO, NO, GSH, and the activities of antioxidant enzymes (SOD, CAT, GR, and GPx) in the brain homogenate of rats. Our findings showed that exposure to Cd (6.5 mg/kg body weight) for 5 consecutive days induced neuronal alterations through the depletion of antioxidant defense mechanisms, which disturbs the cellular redox and leads to oxidative stress. This finding was confirmed by an increase in the levels of LPO and NO and a decrease in the GSH levels and the activities of SOD, CAT, GR, and GPx. The decrease in the levels of antioxidant enzymes may be attributed to the accumulation of Cd in the brain tissue which leads to the consumption of the GSH pool [45]. Depletion in the GSH content inactivates the antioxidant enzymes; in addition, Cd binds to the sulphydryl (–SH) group of the oxidative enzymes, which leads to their inhibition [11]. Treatment with SME significantly restored the balance between the production of ROS and the antioxidant system through the inhibition of the LPO, NO production, and an improvement in the oxidant/antioxidant status in the brain homogenate. High levels of flavonoids in the SME act as a chelator for Cd [41].

The effect of SME on neuronal antioxidant enzymes in Cd-intoxicated rats was determined by measuring the transcriptional levels of *SOD2, CAT, GPx1*, and *GR*. Our results showed down-regulation of all these genes compared with their control levels, which may be because of production of ROS after Cd exposure. The pre-administration of SME up-regulates the expression of the examined antioxidant genes in the brain tissue. Overexpression of antioxidant genes provides neuroprotection against the consequence of oxidative stress [46].

Heavy metals have been widely associated with severe histopathological alterations in the brain tissue [47]. Histological examination of the brain tissue indicates that Cd intoxication causes abnormal structural changes in the brain tissue, including apoptosis, nuclear vacuolization, and lymphocytic inflammatory changes. Once inside the brain, Cd changes the cortical micro- and macrostructures. In the hippocampus, Cd destroys the neurones and glial cells in the white matter [48].

Cd induces neuropathological and neurochemical alterations in the brain, which causes severe damage, including encephalopathy, peripheral neuropathy, and hemorrhage. Moreover, the exposition to Cd alters the cortical neuroglia and pyramidal and granule cells [49]. In addition, Cd decreases attention, impairs memory and olfactory functions, and hypernociception by affecting the structure of nerve cells and parenchyma [50]. Furthermore, the cells are distorted and shrunken because of damage to the structural and functional biosynthesis of cellular proteins, nucleic acids, certain enzymes, and various neurotransmitters [49].

A previous study has shown that Cd leads to apoptosis by affecting the calcium homeostasis in different cells by suppressing calcium-dependent ATPase or by stimulating the inositol triphosphate pathway or interfering with protein kinase C (PKC), mitogen-activated protein kinase, and phospholipase C [51]. qRT-PCR analysis showed an up-regulation in the expression of *Bax* (an apoptosis-inducing gene) and down-regulation in the expression of *Bcl-2* (an apoptosis-inhibiting gene) in the brain tissue. This finding may be attributed to the ability of Cd to enhance the influx of Ca^2+^ into the mitochondria, which disrupts the normal metabolism of mitochondria leading to apoptosis and growth arrest in neuronal cells [52,53]. Rats pretreated with SME suppressed apoptosis in the brain tissue, which suggests that SME increases neuronal viability via the inhibition of Bax-induced apoptosis.

Liu et al. [54] showed that the level of serum and hepatic TNF-α increased after exposure of mice to different doses of Cd in the drinking water for 21 days. Moreover, the i.p. injection of Cd (1 mg of Cd/kg) increased the protein expression of TNF-α in the splenic tissue of rats [55]. In addition, the expression of TNF-α in rat testis increased after injection with CdSO_4_ (3 mg/kg body weight) after 12, 24, and 48 h. Treatment with different strawberry extracts showed anti-inflammatory effects in different experimental models. Oral administration of strawberry leaf extract decreases the serum levels of TNF-α in a rat model of diabetic nephropathy [56]. Oral administration of different strawberry extracts down-regulated the expression of TNF-α in nicotinamide–streptozotocin-induced diabetes in rats [57]. Furthermore, we previously showed that the methanolic extract of strawberry effectively attenuated cadmium-induced inflammation in liver [21], kidney [5], and testis [22] tissues. Our findings showed that compared with Cd-intoxicated rats, rats treated with SME showed down-regulation of TNF-α expression in the brain tissue.

The present study also has limitations, as we administered SME before Cd and to prove the chelation effect of strawberry fruit, we should administer SME post to cadmium and measure Cd concentration in both brain and urine to confirm the ability of SME to clear Cd from the brain and enhance its excretion through the urine. However, many flavonoids such as those in SME can interact with metal and lead to chelation formation.

Overall, our results showed that the SME exerts protective effects against Cd-mediated brain injury through its potent antioxidant, anti-apoptotic, and anti-inflammatory activities, and these effects may be attributed to the high levels of flavonoids and polyphenols in the SME. However, more experiment will be necessary to confirm the biological activities of strawberry on neuronal tissues.

## Supporting information

**Table S1:** Identification of compounds by GC-MS in Fragaria ananassa methanolic extract.

## Abbreviations

CAT: catalase
GAPDH: glyceraldehyde-3-phosphate dehydrogenase
GR: glutathione reductase
GSH: glutathione
i.p: intraperitoneally
LPO: lipid peroxidation
MDA: malondialdehyde
NIH: National Institutes of Health
ROS: reactive oxygen species
SME: *Fragaria ananassa* methanolic extract
SOD: superoxide dismutase
TNF-α: tumor necrosis factor α
