# Expression of Concern: Protective effects of Fragaria ananassa methanolic extract in a rat model of cadmium chloride-induced neurotoxicity

**DOI:** 10.1042/BSR-20180861_EOC

**Published:** 2021-03-26

**Keywords:** apoptosis, brain, cadmium, Fragaria ananassa, oxidative stress

The Editorial Office has been made aware of potential issues surrounding the scientific validity of this paper, hence has issued an expression of concern to notify readers whilst the Editorial Office investigates.

