# Correction: Protective effects of *Fragaria ananassa* methanolic extract in a rat model of cadmium chloride-induced neurotoxicity

**DOI:** 10.1042/BSR-20180861_COR

**Published:** 2021-04-27

**Keywords:** apoptosis, brain, cadmium, Fragaria ananassa, oxidative stress

This Correction follows an Expression of Concern relating to this article previously published by Portland Press.

The authors of the original article “Protective effects of *Fragaria ananassa* methanolic extract in a rat model of cadmium chloride-induced neurotoxicity” (*Biosci Rep* (2018) 38(6), **DOI:** 10.1042/BSR20180861) would like to correct Figure 9, which had been identified as containing overlapping images in two panels.

**Figure 9:**
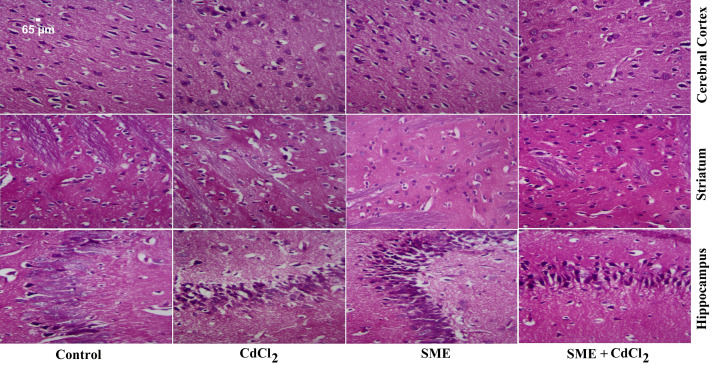
Effect of SME on the histological changes caused by CdCl_2_ injection in the brain of rats Sections of control rats show normal architecture in the different parts of brain. Sections from the CdCl_2_-intoxicated group show extensive neuronal damage, apoptotic neurones, and degeneration of Purkinje neurones. Sections of the SME-treated group show a normal structure. Sections of brains where SME was pre-administered to CdCl_2_ show improvement in the neuronal structure (H&E, ×400).

The authors have stated that the overlapping images had been selected from the same group in error, and further confirm that this error does not affect the conclusions of their study. The correct version of Figure 9 is presented in this Correction.

