# Supplementary material for: Protective effects of Fragaria ananassa methanolic extract in a rat model of cadmium chloride-induced neurotoxicity
Source: Biosci Rep. 2018 Nov 16;38(6):BSR20180861. doi: 10.1042/BSR20180861 (PMC6240722; doi:10.1042/BSR20180861)
Supplement: Supplementary file 1 [file bsr20180861_Supp1.pdf]

Table S1: Identification of compounds by GC-MS in *Fragaria ananassa* methanolic extract.

No.	RT (min)	Name	AUP (%)
1	8.7095	7H-Dibenzo[c,h]phenothiazine	0.1083
2	8.9485	2-Azetidinone, 1-(t-butyldimethylsilyl)-4-[(t-butyldimethylsiloxy)methyl]-3-methylene-	0.0408
3	10.0851	Malic acid	0.5309
4	10.9302	D-(-)-Lyxofuranose, tetrakis(trimethylsilyl) ether	0.0299
5	11.0002	1,2,4,5-Cyclohexanetetrol	0.0619
6	11.4199	D-(+)-Galacturonic acid	0.113
7	11.484	5-Amino-1-(2-chlorophenyl)-2-phenyl-1,2-dihydro-3H-pyrrole-3,3,4-tricarbonitrile	0.019
8	11.7113	L-Rhamnose	0.2664
9	11.7929	D-Psicose, pentakis(trimethylsilyl) ether, pentafluorobenzyloxime	0.0261
10	11.9619	Per-O-(trimethylsilyl)-.alpha.-l-idofuranuronic acid	15.3011
11	12.0202	D-(-)-Fructofuranose, pentakis(trimethylsilyl) ether	35.6698
12	12.0552	D-(-)-Fructopyranose	0.559
13	12.1601	D-(+)-Talofuranose, pentakis(trimethylsilyl) ether	2.9154
14	12.2184	Gulonic acid, .gamma.-lactone	0.0799
15	12.2533	Cyanuric acid	0.1482
16	12.434	alpha.-D-(-)-Ribopyranose	17.8184
17	12.5448	Bucindolol	1.1132
18	12.8071	L-(-)-Sorbofuranose, pentakis(trimethylsilyl) ether	0.0147
19	12.8595	D-Glucose	22.8746
20	14.9345	1,5-Anhydroglucitol	0.181
21	14.9928	D-(-)-Tagatofuranose, pentakis(trimethylsilyl) ether	0.0651
22	15.0453	Quininic acid	0.0305
23	15.2784	Sucrose	0.1183
24	15.4358	Indole-3-carboxaldehyde	0.0203
25	15.4591	3-.alpha.-Mannobiose, octakis(trimethylsilyl) ether	0.0286
26	15.5349	Maltose, octakis(trimethylsilyl) ether, methyloxime	0.184
27	15.5815	Methyl 2-hydroxydocosanoate	0.0463
28	15.6107	3,4-Dihydroxycinnamyl alcohol,tris(O-trimethylsilyl)-	0.0504
29	15.6456	4-Acetyl-6-methoxy-2(1H)-quinolinone	0.1088
30	15.7564	Sucrose	0.8854
31	16.042	5.alpha.-Dihydrotestosterone	0.0606
32	16.1236	2-.alpha.-Mannobiose, octakis(trimethylsilyl) ether, methyloxime	0.0899
33	16.1935	D-(-)-Fructofuranose, pentakis(trimethylsilyl) ether	0.1387
34	16.3451	Prosta-10,13-dien-1-oic acid, 9-(methoxyimino)-15-[(trimethylsilyl)oxy]-, methyl ester, (13E,15S)-	0.0849
35	16.4849	1-Monolinolein	0.0402
36	16.7472	3-.alpha.-Mannobiose, octakis(trimethylsilyl) ether	0.0354
37	16.9571	Ursolic acid	0.0335

38	17.0503	Cholesterol	0.0221
39	17.0736	Indeno[1, 2-b]quinoline, 3-methyl-11-phenylimino-	0.0149
40	17.1028	1H-Indole-2,3-dione, 1-(tert-butyldimethylsilyl)-5-ethyl-, 3-[O-(tert-butyldimethylsilyl)oxime]	0.0266
41	17.1261	Coumarin-3-carboxamide, 8-methoxy-N-(3-chlorophenyl)-	0.044

RT, retention time; AUP, area under peak.
